# Design, Synthesis, and Antiviral Evaluation of Novel 3,4-Dihydropyrimidin-2(1*H*)-one Derivatives

**DOI:** 10.3390/microorganisms14061220

**Published:** 2026-05-28

**Authors:** Chen Yao, Zhi-Cheng Li, Ruo-Hang Li, Peng-Xiang Liu, Hong-Yun Yang, Hang Liu, Bo Ding, Heng Wang, He-Ping Li, Yue-Ying Wang, Sheng-Li Ming, Li-Jun Shi, Meng-Di Wang

**Affiliations:** 1 College of Veterinary Medicine, Henan Agricultural University, Zhengzhou 450046, China; yaochenbiochem@163.com (C.Y.); lzc17839106539@163.com (Z.-C.L.); 18738153405@163.com (R.-H.L.); nrzddefonr81@outlook.com (P.-X.L.); yanghy0227@163.com (H.-Y.Y.); 19544513373@163.com (H.L.); 18737800617@163.com (B.D.); wanghheng9264@163.com (H.W.); liheping1972@126.com (H.-P.L.); wangyueying2008@126.com (Y.-Y.W.); mingsl911102@163.com (S.-L.M.); 2 Key Laboratory of Animal Biochemistry and Nutrition, Ministry of Agriculture and Rural Affairs, Zhengzhou 450046, China; 3 Key Laboratory of Animal Growth and Development of Henan Province, Henan Agricultural University, Zhengzhou 450046, China; 4 College of Sciences, Henan Agricultural University, Zhengzhou 450046, China; 5 College of Life Sciences, Henan University of Animal Husbandry and Economy, Zhengzhou 450046, China

**Keywords:** 4-Dihydropyrimidin-2(1*H*)-one (DHPM), synthesis, antiviral activity, in vivo efficacy

## Abstract

The 3,4-dihydropyrimidin-2(1*H*)-one (DHPM) scaffold possesses diverse biological activities and chemical tunability, allowing structural modifications to modulate antiviral, anticancer, and anti-inflammatory effects. In this study, a series of DHPM derivatives with varied substituents were designed and synthesized, and their structures were characterized by ^1^H NMR, ^13^C NMR and HRMS. Their antiviral activities against pseudorabies virus (PRV) and vesicular stomatitis virus (VSV) were evaluated. In vitro assays revealed that several compounds exhibited significant antiviral effects, with **4bf** (SI = 243.08 against PRV), **4ce** (SI = 196.4 against VSV), and **4be** (SI = 124.2 against PRV; SI = 181.1 against VSV) showing the most potent activity. Further studies demonstrated effective inhibition of viral titers, and Western blot and qRT-PCR analyses confirmed downregulation of viral proteins and related genes. Cytotoxicity tests indicated that **4bf**, **4ce**, and **4be** had CC_50_ values of 270.0, 147.1, and 190.9 μg/mL, respectively, suggesting favorable safety profiles. In vivo experiments showed that **4bf**, without affecting normal growth, alleviated PRV-induced pulmonary inflammation and tissue damage, and improved survival in mice. Taken together, DHPM-based compounds demonstrate promising potential as candidate antiviral agents, warranting further development.

## 1. Introduction

Viral infections continue to pose a major threat to global public health and economic stability. Many viruses are highly transmissible and capable of rapid spread, often leading to severe disease or death and imposing substantial burdens on healthcare systems [1,2,3,4]. In addition, high mutation rates and strong viral adaptability facilitate immune evasion and reduce vaccine effectiveness, which complicates disease control and highlights the limitations of current prophylactic and therapeutic strategies [5,6]. Therefore, the development of small-molecule antiviral agents with broad-spectrum activity, diverse mechanisms of action, and flexible structural design remains an important research priority [7,8].

3,4-Dihydropyrimidin-2(1*H*)-one (DHPM) derivatives represent a class of heterocyclic compounds first synthesized through the multicomponent Biginelli reaction involving aldehydes, β-ketoesters, and urea, which was originally reported by Biginelli in 1893 [9]. The DHPM scaffold is structurally simple yet highly versatile with multiple points for substitution, enabling extensive chemical modification and optimization [10,11,12]. Owing to these features, DHPM derivatives have attracted considerable attention in medicinal chemistry and have been reported to exhibit a wide range of biological activities, including antiviral, antitumor, anti-inflammatory, and antimicrobial effects [13,14,15]. Their antiviral mechanisms may involve inhibition of viral enzymes or proteins [16], interference with virus–host receptor interactions [17,18,19], disruption of viral genome replication, and modulation of host antiviral signaling pathways.

**Figure 1:**
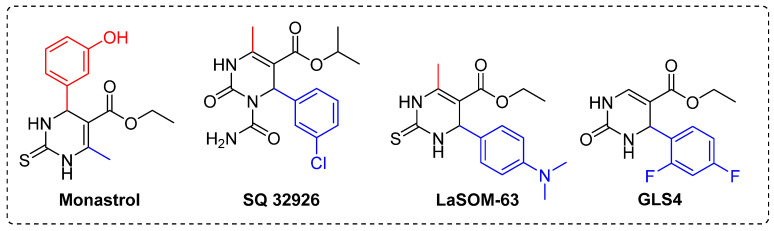
Representative biologically active DHPM derivatives.

Several representative DHPM derivatives have demonstrated notable pharmacological activities [20]. For instance, Monastrol [21] is a well-known inhibitor of the mitotic kinesin Eg5 [22] and has been widely used as a tool compound for investigating cell division. SQ 32926 [23] functions as a calcium channel blocker with potential antihypertensive activity, whereas LaSOM-63 [24] has been reported to possess anti-inflammatory properties [25]. In addition, the antiviral candidate GLS4 acts as a capsid assembly modulator and exhibits potent inhibition of hepatitis B virus replication (Figure 1). These findings highlight the structural versatility and therapeutic potential of DHPM-based compounds, indicating that the DHPM scaffold represents an attractive platform for the development of novel bioactive molecules.

Compared with conventional antiviral agents such as the nucleoside analogues acyclovir and ribavirin, which mainly inhibit viral DNA [26] or RNA [19,27] synthesis, DHPM derivatives provide additional opportunities for multi-target interactions and structural optimization to improve antiviral activity and selectivity [13]. Although several studies have begun to explore the antiviral potential of DHPM analogues, systematic evaluations of their antiviral activity remain relatively limited [28]. In the present study, a series of DHPM derivatives were synthesized using a one-pot strategy in acetic acid, allowing condensation and cyclization to proceed simultaneously without isolation of intermediates. The resulting products were purified by water precipitation followed by methanol recrystallization. This synthetic approach shortens the reaction time, improves yields, and is consistent with green chemistry principles due to reduced solvent consumption and high atom economy [29].

To evaluate antiviral activity, pseudorabies virus (PRV) and vesicular stomatitis virus (VSV) were selected as in vitro infection models. PRV is a highly contagious alphaherpesvirus that infects swine and other mammals [30], causing substantial economic losses and posing potential zoonotic risks [31], while effective small-molecule antivirals remain limited. VSV, a negative-strand RNA virus, is widely used as a model for evaluating broad-spectrum antiviral activity [32,33]. We try to find different types of small-molecule antivirals [32,33,34]. Several synthesized DHPM derivatives significantly inhibited PRV and VSV replication, reduced viral protein expression, and exhibited low cytotoxicity in cell-based assays. Mechanistic investigations further suggest that these compounds may interfere with key steps of viral replication and potentially modulate host antiviral responses. These findings indicate that DHPM derivatives may serve as promising lead compounds for the development of broad-spectrum antiviral agents.

## 2. Materials and Methods

### 2.1. Chemicals and Instruments

All reagents and solvents were purchased from Shanghai Aladdin Biochemical Technology Co., Ltd. (Shanghai, China) and used as received unless otherwise noted.The target compounds were synthesized via a one-pot procedure and characterized by ^1^H and ^13^C NMR and high-resolution mass spectrometry. All NMR spectra were recorded in DMSO-*d*_6_ or CDCl_3_ as solvents. NMR data were obtained on a Bruker DPX-400 Spectrometer (Rheinstetten, Germany), with data processed by MestReNova 14.0.23239 (Santiago de Compostela, Spain) software. ^1^H NMR chemical shifts were referenced to tetramethylsilane (TMS) as the internal standard, and ^13^C NMR chemical shifts were referenced to the deuterated solvent signals. Coupling constants (*J*) are reported in Hz, and chemical shifts (δ) are given in ppm. Signal multiplicities are denoted as s (singlet), d (doublet), t (triplet), q (quartet), and m (multiplet). Unless otherwise specified, each signal corresponds to one proton or one carbon atom. Reaction progress was monitored by thin-layer chromatography (TLC) on plates precoated with fluorescent indicator, and spots were visualized under UV light at 254 and 365 nm.

### 2.2. Chemical Synthesis

Table 1 summarizes all 3,4-dihydropyrimidin-2(1*H*)-one (DHPM) derivatives (**4**) prepared in this study. The detailed synthetic procedures and experimental protocols are provided in Section 2.3.

### 2.3. General Procedure for the Synthesis of DHPM Derivatives ***4aa***–***4cm***

The synthesis of all 3,4-dihydropyrimidin-2(1*H*)-one (DHPM) derivatives in this study are summarized in Table 1. Taking compound **4aa** as an example, the synthetic procedure for this class of compounds is described as follows:

**Table 1:** Preparation for 3,4-dihydropyrimidin-2(1*H*)-one derivatives (**4**).

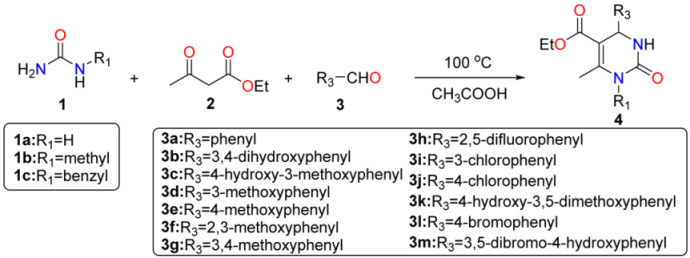
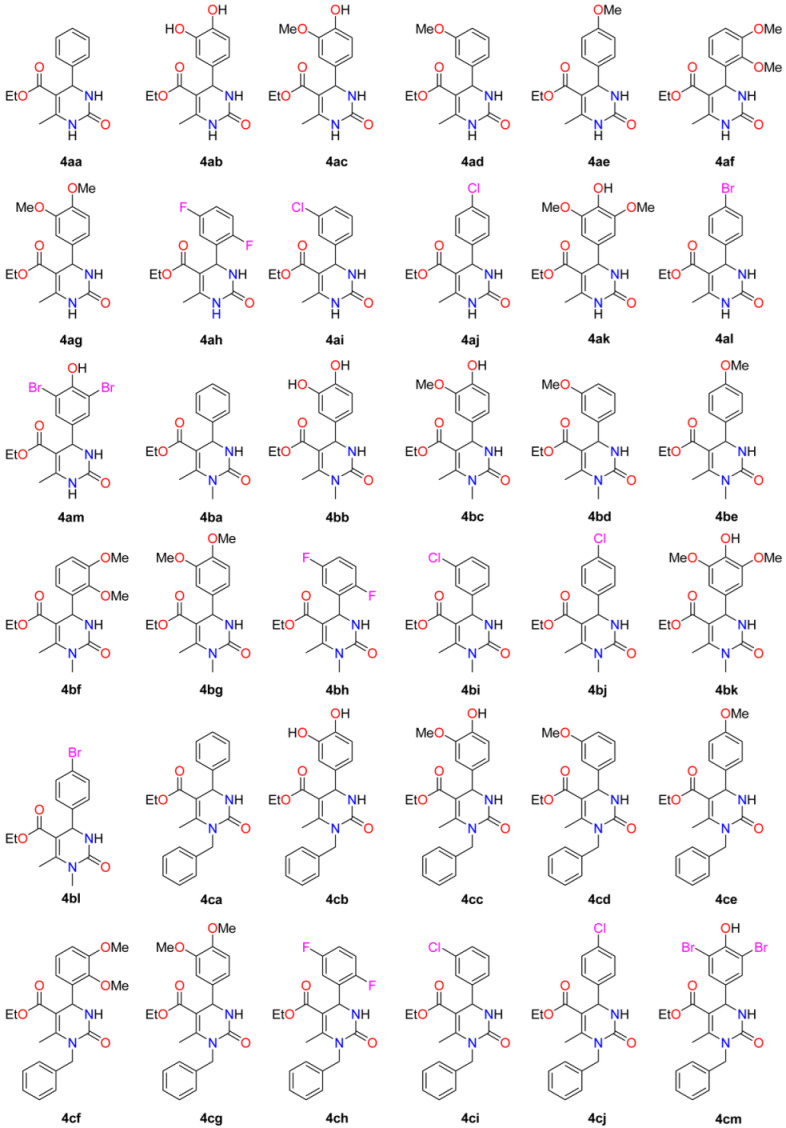

Acetic acid (10 mL) was introduced into a 50 mL round-bottom flask, followed by the sequential addition of urea (**1a**, 0.90 g, 15.0 mmol), benzaldehyde (**3a**, 1.01 mL, 1.06 g, 10.0 mmol), and anhydrous magnesium sulfate (0.36 g, 3.0 mmol). The mixture was stirred thoroughly, after which ethyl acetoacetate (**2**, 1.26 mL, 1.30 g, 10.0 mmol) was added dropwise. The reaction mixture was heated to 100 °C and maintained under continuous stirring for 6–8 h. Reaction progress was monitored by thin-layer chromatography (TLC) using dichloromethane/ethyl acetate (1:1, *v*/*v*) as the eluent. Upon completion, the mixture was allowed to cool to room temperature and then slowly poured into water, leading to the formation of a white precipitate. The resulting solid was collected, washed with water (2–3 times), and recrystallized from methanol to afford **ethyl 6-methyl-2-oxo-4-phenyl-1,2,3,4-tetrahydropyrimidine-5-carboxylate** (**4aa**) as a white solid (2.05 g, 7.85 mmol) in 78.8% yield based on benzaldehyde.

**Ethyl 6-methyl-2-oxo-4-phenyl-1,2,3,4-tetrahydropyrimidine-5-carboxylate** (**4aa**): white solid; 78.8% yield. ^1^H NMR (400 MHz, DMSO-*d*_6_) δ 9.19 (s), 7.75 (s), 7.32 (dd, *J* = 7.9, 6.7 Hz, 2H), 7.28–7.14 (m, 3H), 5.14 (s), 3.98 (q, *J* = 7.0 Hz, 2H), 2.24 (s, 3H), 1.09 (t, *J* = 7.1 Hz, 3H). ^13^C NMR (101 MHz, DMSO-*d*_6_) δ 165.36, 152.16, 148.41, 144.89, 128.43 (2C), 127.30, 126.27 (2C), 99.26, 59.22, 53.97, 17.81, 14.11. HRMS (ESl): caled for C_14_H_16_N_2_O_3_[M+H]^+^: 261.1234, found: 261.1204.

**Ethyl 4-(3,4-dihydroxyphenyl)-1,6-dimethyl-2-oxo-1,2,3,4-tetrahydropyrimidine-5-carboxylate** (**4ab**): yellowish-brown solid; 48.2% yield. ^1^H NMR (400 MHz, DMSO-*d*_6_) δ 9.09 (s), 8.87 (s), 8.78 (s), 7.59 (s), 6.63 (d, *J* = 8.2 Hz, 2H), 6.49 (dd, *J* = 8.1, 2.2 Hz, 1H), 4.97 (d, *J* = 3.4 Hz, 1H), 3.98 (q, *J* = 7.1 Hz, 2H), 2.22 (s, 3H), 1.11 (t, *J* = 7.1 Hz, 3H). ^13^C NMR (101 MHz, DMSO-*d*_6_) δ 165.33, 152.09, 147.37, 144.81, 144.34, 135.85, 117.00, 115.02, 113.55, 99.69, 58.97, 53.35, 17.60, 13.98. HRMS (ESl): caled for C_14_H_16_N_2_O_5_[M+Na]^+^: 315.0951, found: 315.0915.

**Ethyl 4-(4-hydroxy-3-methoxyphenyl)-6-methyl-2-oxo-1,2,3,4-tetrahydropyrimidine-5-carboxylate** (**4ac**): white solid; 49.6% yield. ^1^H NMR (400 MHz, DMSO-*d*_6_) δ 9.12 (s), 8.91 (s), 7.63 (s), 6.79 (d, *J* = 2.0 Hz, 1H), 6.70 (d, *J* = 8.1 Hz, 1H), 6.60 (dd, *J* = 8.2, 2.0 Hz, 1H), 5.05 (d, *J* = 3.2 Hz, 1H), 3.99 (q, *J* = 7.1 Hz, 2H), 3.72 (s, 3H), 2.23 (s, 3H), 1.11 (t, *J* = 7.1 Hz, 3H).^13^C NMR (101 MHz, DMSO-*d*_6_) δ 165.08, 151.88, 147.54, 146.86, 145.39, 135.54, 117.89, 114.88, 110.45, 99.15, 58.76, 55.15, 53.16, 17.37, 13.78. HRMS (ESl): caled for C1_5_H_18_N_2_O_5_[M+H]^+^: 307.1288, found: 307.1254.

**Ethyl 4-(3-methoxyphenyl)-6-methyl-2-oxo-1,2,3,4-tetrahydropyrimidine-5-carboxylate** (**4ad**): white solid; 53.7% yield. ^1^H NMR (400 MHz, DMSO-*d*_6_) δ 9.20 (s), 7.73 (dd, *J* = 3.5, 1.9 Hz, 1H), 7.24 (t, *J* = 7.9 Hz, 1H), 6.82 (d, *J* = 8.1 Hz, 2H), 6.78 (t, *J* = 2.0 Hz, 1H), 5.12 (d, *J* = 3.3 Hz, 1H), 4.00 (q, *J* = 7.1 Hz, 2H), 3.72 (s, 3H), 2.24 (d, *J* = 1.6 Hz, 3H), 1.11 (t, *J* = 7.1 Hz, 3H).^13^C NMR (101 MHz, DMSO-*d*_6_) δ 165.38, 159.23, 152.26, 148.49, 146.37, 129.60, 118.27, 112.43, 112.15, 99.16, 59.26, 54.99, 53.76, 17.80, 14.15. HRMS (ESl): caled for C_15_H_18_N_2_O_4_[M+H]^+^: 291.1339, found: 291.1304.

**Ethyl 4-(4-methoxyphenyl)-6-methyl-2-oxo-1,2,3,4-tetrahydropyrimidine-5-carboxylate** (**4ae**): white solid; 76.5% yield^.1^H NMR (400 MHz, DMSO-*d*_6_) δ 9.16 (s), 7.70–7.64 (m), 7.14 (d, *J* = 8.7 Hz, 2H), 6.87 (d, *J* = 8.7 Hz, 2H), 5.09 (d, *J* = 3.3 Hz, 1H), 3.97 (q, *J* = 7.1 Hz, 2H), 3.71 (s, 3H), 2.24 (s, 3H), 1.10 (t, *J* = 7.1 Hz, 3H).^13^C NMR (101 MHz, DMSO-*d*_6_) δ 165.41, 158.47, 152.20, 148.06, 137.08, 127.43 (2C), 113.73 (2C), 99.58, 59.19, 55.08, 53.36, 17.79, 14.14. HRMS (ESl): caled for C_15_H_18_N_2_O_4_[M+H]^+^: 291.1339, found: 291.1306.

**Ethyl 4-(2,3-dimethoxyphenyl)-6-methyl-2-oxo-1,2,3,4-tetrahydropyrimidine-5-carboxylate** (**4af**): white solid; 64.2% yield. ^1^H NMR (400 MHz, DMSO-*d*_6_) δ 9.15 (s), 7.37 (t, *J* = 2.6 Hz, 1H), 7.03–6.90 (m, 2H), 6.73 (dd, *J* = 7.6, 1.7 Hz, 1H)Hz, 1H), 5.46 (d, *J* = 3.1 Hz, 1H)Hz, 1H), 3.92 (q, *J* = 7.1 Hz, 2H), 3.79 (s, 3H), 3.76 (s, 3H), 2.27 (s, 3H), 1.03 (t, *J* = 7.1 Hz, 3H).^13^C NMR (101 MHz, DMSO-*d*_6_) δ 165.36, 152.48, 151.91, 148.54, 146.09, 137.48, 123.76, 119.55, 112.11, 98.20, 60.13, 59.06, 55.70, 49.54, 17.88, 14.10. HRMS (ESl): caled for C_16_H_20_N_2_O_5_[M+H]^+^: 321.1445, found: 321.1406.

**Ethyl 4-(3,4-dimethoxyphenyl)-6-methyl-2-oxo-1,2,3,4-tetrahydropyrimidine-5-carboxylate** (**4ag**): white solid; 76.5% yield. ^1^H NMR (400 MHz, DMSO-*d*_6_) δ 9.15 (s), 7.67 (dd, *J* = 3.4, 2.0 Hz, 1H), 6.89 (d, *J* = 8.3 Hz, 1H), 6.84 (d, *J* = 2.1 Hz, 1H), 6.72 (dd, *J* = 8.3, 2.1 Hz, 1H), 5.10 (d, *J* = 3.3 Hz, 1H), 4.00 (q, *J* = 7.1 Hz, 2H), 3.71 (s, 6H), 2.24 (s, 3H), 1.12 (t, *J* = 7.1 Hz, 3H).^13^C NMR (101 MHz, DMSO-*d*_6_ δ) 165.43, 152.25, 148.47, 148.16, 148.04, 137.35, 117.89, 111.74, 110.45, 99.36, 59.18, 55.53, 55.41, 53.46, 17.76, 14.17. HRMS (ESl): caled for C_16_H_20_N_2_O_5_[M+H]^+^: 321.1445, found: 321.1408.

**Ethyl 4-(2,5-difluorophenyl)-6-methyl-2-oxo-1,2,3,4-tetrahydropyrimidine-5-carboxylate** (**4ah**): white solid; 45.2% yield. ^1^H NMR (400 MHz, DMSO-*d*_6_) δ 9.32 (s), 7.76 (t, *J* = 2.5 Hz, 1H), 7.28–7.17 (m), 7.20–7.10 (m), 7.06–6.97 (m), 5.40 (d, *J* = 3.0 Hz, 1H), 3.93 (q, *J* = 7.1 Hz, 2H), 2.27 (s, 3H), 1.03 (t, *J* = 7.1 Hz, 3H).^13^C NMR (101 MHz, DMSO-*d*_6_) δ 164.91, 158.09 (d, *J* = 239.5 Hz, 1C), 154.42 (d, *J* = 230.0 Hz, 1C), 151.38, 149.53, 133.54 (dd, *J* = 16.5, 6.2 Hz, 1C), 117.20 (dd, *J* = 25.3, 8.5 Hz, 1C), 115.85 (dd, *J* = 24.1, 8.8 Hz, 1C), 115.05 (dd, *J* = 24.2, 4.6 Hz, 1C), 114.90, 96.77, 59.21, 49.04, 17.80, 13.91. HRMS (ESl): caled for C_14_H_14_F_2_N_2_O_3_[M+Na]^+^: 319.0865, found: 319.0836.

**Ethyl 4-(3-chlorophenyl)-6-methyl-2-oxo-1,2,3,4-tetrahydropyrimidine-5-carboxylate** (**4ai**): white solid; 58.8% yield. ^1^H NMR (400 MHz, DMSO-*d*_6)_ δ 9.29 (s), 7.81 (d, *J* = 2.8 Hz, 2H), 7.37 (t, *J* = 7.7 Hz, 3H), 7.35–7.28 (m, 2H), 7.25 (d, *J* = 1.9 Hz, 2H), 7.20 (dd, *J* = 7.5, 1.6 Hz, 2H), 5.16 (d, *J* = 3.4 Hz, 2H), 4.08–3.91 (m, 2H), 2.26 (s, 3H), 1.09 (t, *J* = 7.1 Hz, 3H).^13^C NMR (101 MHz, DMSO-*d*_6_) δ 165.21, 151.98, 149.03, 147.29, 132.95, 130.54, 127.30, 126.29, 124.95, 98.63, 59.35, 53.66, 17.87, 14.08. HRMS (ESl): caled for C_14_H_15_ClN_2_O_3_[M+H]^+^: 295.0844, found: 295.0816.

**Ethyl 4-(4-chlorophenyl)-6-methyl-2-oxo-1,2,3,4-tetrahydropyrimidine-5-carboxylate** (**4aj**): white solid; 81.6% yield. ^1^H NMR (400 MHz, DMSO-*d*_6_) δ 9.27 (d, *J* = 2.1 Hz, 1H), 7.80 (dd, *J* = 3.5, 2.0 Hz, 1H), 7.39 (d, *J* = 8.4 Hz, 2H), 7.25 (d, *J* = 8.5 Hz, 2H), 5.15 (d, *J* = 3.3 Hz, 1H), 3.98 (q, *J* = 7.1 Hz, 2H), 2.26 (s, 3H), 1.08 (t, *J* = 7.1 Hz, 3H).^13^C NMR (101 MHz, DMSO-*d*_6_) δ 165.27, 152.05, 148.80, 143.86, 131.87, 128.46 (2C), 128.26 (2C), 98.89, 59.33, 53.50, 17.88, 14.11. HRMS (ESl): caled for C_14_H_15_ClN_2_O_3_[M+H]^+^: 295.0844, found: 295.0817.

**Ethyl 4-(4-hydroxy-3,5-dimethoxyphenyl)-6-methyl-2-oxo-1,2,3,4-tetrahydropyrimidine-5-carboxylate** (**4ak**): white solid; 40.1% yield.^1^H NMR (400 MHz, DMSO-*d*_6_) δ 9.14 (s), 8.33 (s), 7.66 (s), 6.48 (s, 2H), 5.07 (d, *J* = 3.3 Hz, 1H), 4.01 (q, *J* = 7.0 Hz, 2H), 3.70 (s, 6H), 2.24 (s, 3H), 1.13 (t, *J* = 7.1 Hz, 3H).^13^C NMR (101 MHz, DMSO-*d*_6_) δ 165.92, 152.71, 148.48 (2C), 148.22, 135.47, 104.22 (2C), 99.82, 59.61, 56.41 (2C), 54.23, 18.18, 14.64. HRMS (ESl): caled for C_16_H_20_N_2_O_6_[M+Na]^+^: 359.1214, found: 359.1178.

**Ethyl 4-(4-bromophenyl)-6-methyl-2-oxo-1,2,3,4-tetrahydropyrimidine-5-carboxylate** (**4al**): white solid; 62.7% yield. ^1^H NMR (400 MHz, DMSO-*d*_6_) δ 9.24 (d, *J* = 2.0 Hz, 1H), 7.77 (dd, *J* = 3.5, 2.0 Hz, 1H), 7.53 (d, *J* = 8.4 Hz, 2H), 7.19 (d, *J* = 8.4 Hz, 2H), 5.12 (d, *J* = 3.3 Hz, 1H), 3.98 (q, *J* = 7.1 Hz, 2H), 2.24 (s, 3H), 1.09 (t, *J* = 7.1 Hz, 3H).^13^C NMR (101 MHz, DMSO-*d*_6_) δ 165.20, 151.93, 148.75, 144.21, 131.33 (2C), 128.56 (2C), 120.31, 98.75, 59.27, 53.48, 17.81, 14.08. HRMS (ESl): caled for C_14_H_15_BrN_2_O_3_[M+Na]^+^: 361.0158, found: 361.0123.

**Ethyl 4-(3,5-dibromo-4-hydroxyphenyl)-6-methyl-2-oxo-1,2,3,4-tetrahydropyrimidine-5-carboxylate** (**4am**): off-white solid; 83.3% yield.^1^H NMR (400 MHz, DMSO-*d*_6_) δ 9.96 (s), 9.28 (d, *J* = 2.0 Hz, 1H), 7.76 (dd, *J* = 3.3, 1.9 Hz,), 7.33 (s, 2H), 5.07 (d, *J* = 3.4 Hz, 1H), 4.10–3.90 (m, 2H), 2.25 (s, 3H), 1.11 (t, *J* = 7.1 Hz, 3H).^13^C NMR (101 MHz, DMSO-*d*_6_) δ 165.18, 151.86, 149.97, 149.01, 139.24, 130.15 (2C), 111.85 (2C), 98.57, 59.41, 52.80, 17.91, 14.12. HRMS (ESl): caled for C_14_H_14_Br_2_N_2_O_4_[M+Na]^+^: 456.9192, found: 456.9147.

**Ethyl 1,6-dimethyl-2-oxo-4-phenyl-1,2,3,4-tetrahydropyrimidine-5-carboxylate** (**4ba**): white solid; 46.6% yield.^1^H NMR (400 MHz, DMSO-*d*_6_) δ 7.99 (d, *J* = 3.9 Hz, 1H), 7.33 (dd, *J* = 8.0, 6.7 Hz, 2H), 7.29–7.20 (m, 3H), 5.18 (d, *J* = 3.8 Hz, 1H), 4.04 (q, *J* = 7.1 Hz, 2H), 3.11 (s, 3H), 1.13 (t, *J* = 7.1 Hz, 3H).^13^C NMR (101 MHz, DMSO-*d*_6_) δ 165.62, 153.12, 150.60, 144.11, 128.49 (2C), 127.35, 126.08 (2C), 102.48, 59.56, 52.44, 29.74, 16.06, 14.08. HRMS (ESl): caled for C_15_H_18_N_2_O_3_[M+H]^+^: 275.1390, found: 275.1362.

**Ethyl 4-(3,4-dihydroxyphenyl)-1,6-dimethyl-2-oxo-1,2,3,4-tetrahydropyrimidine-5-carboxylate** (**4bb**): yellowish-brown solid; 61.4% yield. ^1^H NMR (400 MHz, DMSO-*d*_6_) δ 8.86 (s), 8.79 (s), 7.81 (d, *J* = 3.8 Hz, 1H), 6.67–6.58 (m, 2H), 6.46 (dd, *J* = 8.1, 2.2 Hz, 1H), 4.99 (d, *J* = 3.7 Hz, 1H), 4.03 (q, *J* = 7.1 Hz, 2H), 3.09 (s, 3H), 2.45 (s, 3H), 1.14 (t, *J* = 7.1 Hz, 3H).^13^C NMR (101 MHz, DMSO-*d*_6_) δ 165.78, 153.22, 149.62, 145.02, 144.55, 135.22, 116.87, 115.29, 113.62, 103.23, 59.50, 51.97, 29.72, 16.07, 14.14. HRMS (ESl): caled for C_15_H_18_N_2_O_5_[M+Na]^+^: 329.1108, found: 329.1074.

**Ethyl 4-(4-hydroxy-3-methoxyphenyl)-1,6-dimethyl-2-oxo-1,2,3,4-tetrahydropyrimidine-5-carboxylate** (**4bc**): white solid; 34.1% yield. ^1^H NMR (400 MHz, DMSO-*d*_6_) δ 8.93 (s), 7.86 (d, *J* = 3.8 Hz, 1H), 6.77 (d, *J* = 2.0 Hz, 1H), 6.70 (d, *J* = 8.1 Hz, 1H), 6.59 (dd, *J* = 8.1, 2.0 Hz, 1H), 5.07 (d, *J* = 3.7 Hz, 1H), 4.04 (q, *J* = 7.1 Hz, 2H), 3.72 (s, 3H), 3.09 (s, 3H), 2.47 (s, 3H), 1.13 (t, *J* = 7.1 Hz, 3H).^13^C NMR (101 MHz, DMSO-*d*_6_) δ 165.75, 153.28, 150.10, 147.36, 145.86, 135.12, 118.12, 115.32, 110.56, 102.93, 59.52, 55.53, 52.08, 29.68, 16.04, 14.17. HRMS (ESl): caled for C_16_H_20_N_2_O_5_[M+H]^+^: 321.1445, found: 321.1412.

**Ethyl 4-(3-methoxyphenyl)-1,6-dimethyl-2-oxo-1,2,3,4-tetrahydropyrimidine-5-carboxylate** (**4bd**): white solid; 61.5% yield. ^1^H NMR (400 MHz, DMSO-*d*_6_) δ 7.96 (d, *J* = 3.9 Hz, 1H), 7.23 (t, *J* = 7.9 Hz, 1H), 6.85–6.75 (m, 2H), 6.75 (d, *J* = 2.2 Hz, 1H), 5.13 (d, *J* = 3.8 Hz, 1H), 4.04 (q, *J* = 7.2 Hz, 2H), 3.72 (s, 3H), 3.09 (s, 3H), 2.48 (s, 3H), 1.13 (t, *J* = 7.1 Hz, 3H).^13^C NMR (101 MHz, DMSO-*d*_6_) δ 165.62, 159.26, 153.20, 150.67, 145.59, 129.65, 118.05, 112.20, 112.14, 102.38, 59.59, 54.96, 52.18, 29.73, 16.04, 14.12. HRMS (ESl): caled for C_16_H_20_N_2_O_4_[M+H]^+^: 305.1496, found: 305.1465.

**Ethyl 4-(4-methoxyphenyl)-1,6-dimethyl-2-oxo-1,2,3,4-tetrahydropyrimidine-5-carboxylate** (**4be**): white solid; 46.0% yield. ^1^H NMR (400 MHz, DMSO-*d*_6_) δ 7.91 (d, *J* = 3.8 Hz, 1H), 7.13 (d, *J* = 8.7 Hz, 2H), 6.87 (d, *J* = 8.7 Hz, 2H), 5.10 (d, *J* = 3.7 Hz, 1H), 4.02 (q, *J* = 7.1 Hz, 2H), 3.71 (s, 3H), 3.09 (s, 3H), 2.48 (s, 3H), 1.12 (t, *J* = 7.1 Hz, 3H).^13^C NMR (101 MHz, DMSO-*d*_6_) δ 165.65, 158.50, 153.13, 150.27, 136.21, 127.26 (2C), 113.78 (2C), 102.79, 59.52, 55.06, 51.82, 29.71, 16.04, 14.11. HRMS (ESl): caled for C_16_H_20_N_2_O_4_[M+H]^+^: 305.1496, found: 305.1465.

**Ethyl 4-(2,3-dimethoxyphenyl)-1,6-dimethyl-2-oxo-1,2,3,4-tetrahydropyrimidine-5-carboxylate** (**4bf**): white solid; 52.9% yield.^1^H NMR (400 MHz, DMSO-*d*_6_) δ 7.59 (d, *J* = 3.7 Hz, 1H), 7.03–6.90 (m, 2H), 6.72 (dd, *J* = 7.3, 2.0 Hz, 1H), 5.47 (d, *J* = 3.6 Hz, 1H), 3.97 (q, *J* = 7.1 Hz, 2H), 3.79 (s, 3H), 3.75 (s, 3H), 3.13 (s, 3H), 1.06 (t, *J* = 7.1 Hz, 3H).^13^C NMR (101 MHz, DMSO-*d*_6_) δ 165.65, 152.83, 152.51, 150.37, 146.18, 136.67, 123.75, 119.32, 112.26, 101.59, 60.20, 59.42, 55.68, 48.35, 29.65, 16.16, 14.05. HRMS (ESl): caled for C_17_H_22_N_2_O_5_[M+H]^+^: 335.1601, found: 335.1566.

**Ethyl 4-(3,4-dimethoxyphenyl)-1,6-dimethyl-2-oxo-1,2,3,4-tetrahydropyrimidine-5-carboxylate** (**4bg**): white solid; 35.6% yield.^1^H NMR (400 MHz, DMSO-*d*_6_) δ 7.90 (d, *J* = 3.8 Hz, 1H), 6.87 (d, *J* = 8.3 Hz, 1H), 6.81 (d, *J* = 2.1 Hz, 1H), 6.70 (dd, *J* = 8.3, 2.1 Hz, 1H), 5.10 (d, *J* = 3.8 Hz, 1H), 4.04 (q, *J* = 7.0 Hz, 2H), 3.71 (s, 6H), 3.09 (s, 3H), 2.48 (s, 3H), 1.14 (t, *J* = 7.1 Hz, 3H).^13^C NMR (101 MHz, DMSO-*d*_6_) δ 165.66, 153.24, 150.32, 148.54, 148.06, 136.47, 117.65, 111.69, 110.11, 102.70, 59.51, 55.50, 55.37, 51.89, 29.66, 16.00, 14.14. HRMS (ESl): caled for C_17_H_22_N_2_O_5_[M+H]^+^: 335.1601, found: 335.1566.

**Ethyl 4-(2,5-difluorophenyl)-1,6-dimethyl-2-oxo-1,2,3,4-tetrahydropyrimidine-5-carboxylate** (**4bh**): yellow solid; 59.6% yield.^1^H NMR (400 MHz, DMSO-*d*_6_) δ 7.99 (d, *J* = 3.6 Hz, 1H), 7.26–7.19 (m), 7.18–7.11 (m), 7.08–6.99 (m), 5.44 (d, *J* = 3.5 Hz, 1H), 3.96 (q, *J* = 7.1 Hz, 2H), 3.15 (s, 3H), 2.53 (s, 3H), 1.05 (t, *J* = 7.1 Hz, 3H).^13^C NMR (101 MHz, DMSO-*d*_6_) δ 165.16, 155.59 (dd, *J* = 242.6, 2.2 Hz, 1C), 132.90 (dd, *J* = 16.8, 6.2 Hz, 1C), 152.33, 151.80, 132.90 (dd, *J* = 16.8, 6.2 Hz, 1C), 117.36, 117.19 (dd, *J* = 25.4, 8.6 Hz, 1C), 115.98 (dd, *J* = 24.1, 8.8 Hz, 1C), 114.83 (dd, *J* = 24.1, 4.6 Hz, 1C), 99.93, 59.54, 47.49, 47.46, 29.66, 16.07, 13.84. HRMS (ESl): caled for C_15_H_16_F_2_N_2_O_3_[M+H]^+^: 311.1202, found: 311.1171.

**Ethyl 4-(3-chlorophenyl)-1,6-dimethyl-2-oxo-1,2,3,4-tetrahydropyrimidine-5-carboxylate** (**4bi**): white solid; 40.2% yield.^1^H NMR (400 MHz, DMSO-*d*_6_) δ 8.05 (d, *J* = 3.9 Hz, 1H), 7.33 (m, 2H), 7.26 (d, *J* = 2.0 Hz, 1H), 7.20 (dd, *J* = 7.5, 1.7 Hz, 1H), 5.19 (d, *J* = 3.7 Hz, 1H), 4.04 (q, *J* = 7.1 Hz, 2H), 3.11 (s, 3H), 1.11 (t, *J* = 7.1 Hz, 3H).^13^C NMR (101 MHz, DMSO-*d*_6_) δ 165.46, 152.91, 151.30, 146.57, 133.08, 130.52, 127.35, 126.19, 124.76, 101.71, 59.65, 52.15, 29.77, 16.09, 14.04. HRMS (ESl): caled for C_15_H_17_ClN_2_O_3_[M+H]^+^: 309.1000, found: 309.0970.

**Ethyl 4-(4-chlorophenyl)-1,6-dimethyl-2-oxo-1,2,3,4-tetrahydropyrimidine-5-carboxylate** (**4bj**): white solid; 42.2% yield.^1^H NMR (400 MHz, DMSO-*d*_6_) δ 8.03 (d, *J* = 3.9 Hz, 1H), 7.39 (d, *J* = 8.5 Hz, 2H), 7.25 (d, *J* = 8.5 Hz, 2H), 5.17 (d, *J* = 3.8 Hz, 1H), 4.03 (q, *J* = 7.1 Hz, 2H), 3.11 (s, 3H), 1.12 (t, *J* = 7.1 Hz, 3H).^13^C NMR (101 MHz, DMSO-*d*_6_) δ 165.48, 152.93, 151.05, 143.06, 131.93, 128.48 (2C), 128.06 (2C), 101.98, 59.63, 51.92, 29.77, 16.07, 14.07. HRMS (ESl): caled for C_15_H_17_ClN_2_O_3_[M+H]^+^: 309.1000, found: 309.0969.

**Ethyl 4-(4-hydroxy-3,5-dimethoxyphenyl)-1,6-dimethyl-2-oxo-1,2,3,4-tetrahydropyrimidine-5-carboxylate** (**4bk**): white solid; 50.5% yield. ^1^H NMR (400 MHz, DMSO-*d*_6_) δ 8.34 (s), 7.88 (s), 6.44 (s, 2H), 5.07 (s), 4.05 (q, *J* = 7.1 Hz, 2H), 3.69 (s, 6H), 3.10 (s, 3H), 2.47 (s, 3H), 1.15 (t, *J* = 7.1 Hz, 3H).^13^C NMR (101 MHz, DMSO-*d*_6_) δ 165.73, 153.36, 150.20, 147.83 (2C), 134.97, 134.17, 103.47 (2C), 102.88, 59.53, 55.95 (2C), 52.22, 29.65, 16.01, 14.20. HRMS (ESl): caled for C_17_H_22_N_2_O_6_[M+H]^+^: 351.1551, found: 351.1515.

**Ethyl 4-(4-bromophenyl)-1,6-dimethyl-2-oxo-1,2,3,4-tetrahydropyrimidine-5-carboxylate** (**4bl**): white solid; 35.5% yield. ^1^H NMR (400 MHz, DMSO-*d*_6_) δ 8.00 (d, *J* = 3.9 Hz, 1H), 7.52 (d, *J* = 8.4 Hz, 2H), 7.17 (d, *J* = 8.5 Hz, 2H), 5.14 (d, *J* = 3.8 Hz, 1H), 4.03 (q, *J* = 7.1 Hz, 2H), 3.09 (s, 3H), 2.49 (s, 3H), 1.11 (t, *J* = 7.1 Hz, 3H).^13^C NMR (101 MHz, DMSO-*d*_6_ δ 165.43, 152.87, 151.01, 143.42, 131.36 (2C), 128.37 (2C), 120.40, 101.89, 59.59, 51.91, 29.74, 16.05, 14.05. HRMS (ESl): caled for C_15_H_17_BrN_2_O_3_[M+Na]^+^: 375.0315, found: 375.0278.

**Ethyl 1-benzyl-6-methyl-2-oxo-4-phenyl-1,2,3,4-tetrahydropyrimidine-5-carboxylate** (**4ca**). White solid; yield 43.0%; ^1^H NMR (400 MHz, DMSO-*d*_6_) δ 8.17 (d, *J* = 3.6 Hz, 1H), 7.37–7.20 (m, 8H), 7.08 (d, *J* = 6.6 Hz, 2H), 5.26 (d, *J* = 3.6 Hz, 1H), 5.11 (d, *J* = 16.8 Hz, 1H), 4.85 (d, *J* = 16.9 Hz, 1H), 4.02 (q, *J* = 7.1 Hz, 2H), 2.37 (s, 3H), 1.09 (t, *J* = 7.2 Hz, 3H).^13^C NMR (101 MHz, DMSO-*d*_6_) δ 165.61, 153.08, 149.46, 143.95, 138.69, 128.50, 128.43 (2C), 127.44, 126.87 (2C), 126.22 (2C), 126.17 (2C), 103.56, 59.72, 52.53, 44.91, 16.00, 14.02. HRMS (ESl): caled for C_21_H_22_N_2_O_3_[M+H]^+^: 351.1703, found: 351.1667.

**Ethyl 1-benzyl-4-(3,4-dihydroxyphenyl)-6-methyl-2-oxo-1,2,3,4-tetrahydropyrimidine-5-carboxylate** (**4cb**). Yellow solid; yield 45.0%;^1^H NMR (400 MHz, DMSO-*d*_6_) δ 8.90 (s), 8.85 (s), 8.00 (d, *J* = 3.6 Hz, 1H), 7.36–7.17 (m, 3H), 7.08 (d, *J* = 6.7 Hz, 2H), 6.73–6.60 (m, 2H), 6.49 (dd, *J* = 8.1, 2.1 Hz, 1H), 5.19–5.01 (m, 2H), 4.84 (d, *J* = 16.9 Hz, 1H), 4.02 (q, *J* = 7.1 Hz, 2H), 2.31 (s, 3H), 1.12 (t, *J* = 7.1 Hz, 3H).^13^C NMR (101 MHz, DMSO-*d*_6_) δ 165.81, 153.18, 148.55, 145.07, 144.61, 138.83, 135.04, 128.51 (2C), 126.79, 126.07 (2C), 116.94, 115.29, 113.86, 104.16, 59.67, 52.03, 44.92, 15.96, 14.10. HRMS (ESl): caled for C_15_H_17_BrN_2_O_3_[M+Na]^+^: 405.1421, found: 405.1380.

**Ethyl 1-benzyl-4-(4-hydroxy-3-methoxyphenyl)-6-methyl-2-oxo-1,2,3,4-tetrahydropyrimidine-5-carboxylate** (**4cc**). White solid; yield 61.3%;^1^H NMR (400 MHz, DMSO-*d*_6_) δ 8.98 (s), 8.04 (d, *J* = 3.6 Hz, 1H), 7.31–7.22 (m, 3H), 7.10 (d, *J* = 7.3 Hz, 2H), 6.80 (d, *J* = 2.1 Hz, 1H), 6.72 (d, *J* = 8.1 Hz, 1H), 6.62 (dd, *J* = 8.1, 2.0 Hz, 1H), 5.17 (d, *J* = 3.5 Hz, 1H), 5.12 (d, *J* = 16.7 Hz, 1H), 4.82 (d, *J* = 16.8 Hz, 1H), 4.03 (q, *J* = 7.1 Hz, 2H), 3.69 (s, 3H), 2.36 (s, 3H), 1.11 (t, *J* = 7.1 Hz, 3H).^13^C NMR (101 MHz, DMSO-*d*_6_) δ 165.78, 153.12, 148.86, 147.43, 145.96, 138.83, 135.00, 128.52 (2C), 126.88, 126.18 (2C), 118.45, 115.25, 110.65, 103.79, 59.68, 55.54, 52.32, 44.85, 16.01, 14.11. HRMS (ESl): caled for C_22_H_24_N_2_O_5_[M+H]^+^: 397.1758, found: 397.1717.

**Ethyl 1-benzyl-4-(3-methoxyphenyl)-6-methyl-2-oxo-1,2,3,4-tetrahydropyrimidine-5-carboxylate** (**4cd**). White solid; yield 47.8%; ^1^H NMR (400 MHz, DMSO-*d*_6_) δ 8.15 (d, *J* = 3.7 Hz, 1H), 7.31–7.20 (m, 4H), 7.07 (d, *J* = 6.9 Hz, 2H), 6.88–6.78 (m, 3H), 5.23 (d, *J* = 3.6 Hz, 1H), 5.11 (d, *J* = 16.8 Hz, 1H), 4.83 (d, *J* = 16.8 Hz, 1H), 4.04 (q, *J* = 7.1 Hz, 2H), 3.71 (s, 3H), 2.36 (s, 3H), 1.11 (t, *J* = 7.1 Hz, 3H).^13^C NMR (101 MHz, DMSO-*d*_6_) δ 165.62, 159.33, 153.13, 149.52, 145.48, 138.69, 129.60, 128.50 (2C), 126.85, 126.11 (2C), 118.25, 112.40, 112.24, 103.39, 59.75, 54.99, 52.33, 44.88, 15.99, 14.06. HRMS (ESl): caled for C_22_H_24_N_2_O_4_[M+H]^+^: 381.1809, found: 381.1769.

**Ethyl 1-benzyl-4-(4-methoxyphenyl)-6-methyl-2-oxo-1,2,3,4-tetrahydropyrimidine-5-carboxylate** (**4ce**). White solid; yield 39.4%; ^1^H NMR (400 MHz, DMSO-*d*_6_) δ 8.09 (d, *J* = 3.6 Hz, 1H), 7.28 (d, *J* = 7.7 Hz, 2H), 7.25 (d, *J* = 1.3 Hz, 1H), 7.14 (d, *J* = 8.6 Hz, 2H), 7.07 (d, *J* = 6.9 Hz, 2H), 6.88 (d, *J* = 8.7 Hz, 2H), 5.18 (d, *J* = 3.6 Hz, 1H), 5.08 (d, *J* = 16.8 Hz, 1H), 4.84 (d, *J* = 16.8 Hz, 1H), 4.01 (q, *J* = 7.3 Hz, 2H), 3.73 (s, 3H), 2.35 (s, 3H), 1.10 (t, *J* = 7.1 Hz, 3H).^13^C NMR (101 MHz, DMSO-*d*_6_) δ 165.65, 158.57, 153.05, 149.11, 138.74, 136.02, 128.52 (2C), 127.41 (2C), 126.87, 126.15 (2C), 113.73 (2C), 103.80, 59.68, 55.12, 51.94, 44.88, 15.98, 14.06. HRMS (ESl): caled for C_22_H_24_N_2_O_4_[M+H]^+^: 381.1809, found: 381.1770.

**Ethyl 1-benzyl-4-(2,3-dimethoxyphenyl)-6-methyl-2-oxo-1,2,3,4-tetrahydropyrimidine-5-carboxylate** (**4cf**). Off-white solid; yield 45.8%;^1^H NMR (400 MHz, DMSO-*d*_6_) δ 7.73 (d, *J* = 3.4 Hz, 1H), 7.33 (d, *J* = 7.4 Hz, 2H), 7.25 (t, *J* = 7.3 Hz, 1H), 7.19 (d, *J* = 8.8 Hz, 2H), 6.97 (d, *J* = 3.9 Hz, 2H), 6.69 (d, *J* = 4.1 Hz, 1H), 5.53 (s), 5.08–4.84 (m, 2H), 4.03–3.84 (m, 2H), 3.80 (s, 3H), 3.74 (s, 3H), 2.36 (s, 3H), 1.03 (t, *J* = 7.1 Hz, 3H).^13^C NMR (101 MHz, DMSO-*d*_6_) δ 165.61, 152.71, 152.50, 149.01, 146.17, 138.93, 136.51, 128.52 (2C), 126.88, 126.29 (2C), 123.65, 119.36, 112.37, 102.35, 60.18, 59.53, 55.71, 48.64, 45.10, 16.08, 13.97. HRMS (ESl): caled for C_23_H_26_N_2_O_5_[M+Na]^+^: 433.1734, found: 433.1693.

**Ethyl 1-benzyl-4-(3,4-dimethoxyphenyl)-6-methyl-2-oxo-1,2,3,4-tetrahydropyrimidine-5-carboxylate** (**4cg**). White solid; yield 18.7%;^1^H NMR (400 MHz, DMSO-*d*_6_) δ 8.08 (d, *J* = 3.7 Hz, 1H), 7.26 (ddd, *J* = 14.4, 7.8, 6.1 Hz, 3H), 7.09 (d, *J* = 8.9 Hz, 2H), 6.89 (d, *J* = 8.3 Hz, 1H), 6.84 (d, *J* = 2.1 Hz, 1H), 6.73 (dd, *J* = 8.3, 2.1 Hz, 1H), 5.20 (d, *J* = 3.5 Hz, 1H), 5.11 (d, *J* = 16.8 Hz, 1H), 4.82 (d, *J* = 16.9 Hz, 1H), 4.03 (q, *J* = 7.1 Hz, 2H), 3.74 (s, 3H), 3.67 (s, 3H), 2.36 (s, 3H), 1.12 (t, *J* = 7.1 Hz, 3H).^13^C NMR (101 MHz, DMSO-*d*_6_) δ 165.67, 153.07, 149.10, 148.58, 148.16, 138.75, 136.37, 128.48 (2C), 126.84, 126.10 (2C), 118.03, 111.62, 110.20, 103.54, 59.66, 55.56, 55.37, 52.14, 44.81, 15.96, 14.08. HRMS (ESl): caled for C_23_H_26_N_2_O_5_[M+Na]^+^: 433.1734, found: 433.1693.

**Ethyl 1-benzyl-4-(2,5-difluorophenyl)-6-methyl-2-oxo-1,2,3,4-tetrahydropyrimidine-5-carboxylate** (**4ch**). White solid; yield 70.9%;^1^H NMR (400 MHz, DMSO-*d*_6_) δ 8.19 (d, *J* = 3.4 Hz, 1H), 7.34 (t, *J* = 7.5 Hz, 2H), 7.28–7.13 (m, 5H), 7.04–6.92 (m), 5.54 (d, *J* = 3.3 Hz, 1H), 5.19–4.83 (m, 2H), 3.95 (q, *J* = 7.1 Hz, 2H), 2.43 (s, 3H), 1.02 (t, *J* = 7.1 Hz, 3H).^13^C NMR (101 MHz, DMSO-*d*_6_) δ 165.10, 158.20 (dd, *J* = 240.9, 2.0 Hz, 1C), 155.62 (dd, *J* = 242.7, 2.2 Hz, 1C), 152.30, 150.54, 138.72, 132.80 (dd, *J* = 16.7, 6.2 Hz, 1C), 128.60 (2C), 127.00, 126.29 (2C), 117.28 (dd, *J* = 25.2, 8.7 Hz, 1C), 116.08 (dd, *J* = 24.1, 8.8 Hz, 1C), 114.85 (dd, *J* = 24.4, 4.5 Hz, 1C), 100.82, 59.69, 47.77, 47.74, 45.21, 16.04, 13.77. HRMS (ESl): caled for C_21_H_20_F_2_N_2_O_3_[M+H]^+^: 387.1515, found: 387.1476.

**Ethyl 1-benzyl-4-(3-chlorophenyl)-6-methyl-2-oxo-1,2,3,4-tetrahydropyrimidine-5-carboxylate** (**4ci**). White solid; yield 40.8%; ^1^H NMR (400 MHz, DMSO-*d*_6_) δ 8.24 (d, *J* = 3.7 Hz, 1H), 7.36 (d, *J* = 6.9 Hz, 2H), 7.28 (dd, *J* = 5.0, 2.6 Hz, 3H), 7.25–7.20 (m, 2H), 7.08 (d, *J* = 6.8 Hz, 2H), 5.27 (d, *J* = 3.6 Hz, 1H), 5.15 (d, *J* = 16.8 Hz, 1H), 4.83 (d, *J* = 16.8 Hz, 1H), 4.10–3.96 (m, 2H), 2.39 (s, 3H), 1.09 (t, *J* = 7.1 Hz, 3H).^13^C NMR (101 MHz, DMSO-*d*_6_) δ 165.41, 152.85, 150.12, 146.41, 138.58, 133.14, 130.48, 128.58 (2C), 127.46, 126.93 (2C), 126.16, 125.00, 102.81, 59.82, 52.20, 44.89, 16.05, 13.99. HRMS (ESl): caled for C_21_H_21_ClN_2_O_3_[M+Na]^+^: 407.1133, found: 407.1091.

**Ethyl 1-benzyl-4-(4-chlorophenyl)-6-methyl-2-oxo-1,2,3,4-tetrahydropyrimidine-5-carboxylate** (**4cj**). White solid; yield 53.3%; ^1^H NMR (400 MHz, DMSO-*d*_6_) δ 8.21 (d, *J* = 3.8 Hz, 1H), 7.40 (d, *J* = 8.4 Hz, 2H), 7.33–7.19 (m, 5H), 7.05 (d, *J* = 6.9 Hz, 2H), 5.23 (d, *J* = 3.7 Hz, 1H), 5.08 (d, *J* = 16.8 Hz, 1H), 4.85 (d, *J* = 16.8 Hz, 1H), 4.02 (q, *J* = 7.1 Hz, 2H), 2.37 (s, 3H), 1.09 (t, *J* = 7.1 Hz, 3H).^13^C NMR (101 MHz, DMSO-*d*_6_) δ 165.45, 152.88, 149.90, 142.91, 138.59, 132.00, 128.52 (2C), 128.42 (2C), 128.16 (2C), 126.91, 126.13, (2C) 103.10, 59.79, 51.94, 44.93, 16.02, 14.02. HRMS (ESl): caled for C_21_H_21_ClN_2_O_3_[M+Na]^+^: 407.1133, found: 407.1091.

**Ethyl 1-benzyl-4-(3,5-dibromo-4-hydroxyphenyl)-6-methyl-2-oxo-1,2,3,4-tetrahydropyrimidine-5-carboxylate** (**4cm**). Off-white solid; yield 40.1%; ^1^H NMR (400 MHz, DMSO-*d*_6_) δ 10.02 (s), 8.17 (d, *J* = 3.6 Hz, 1H), 7.37 (s, 2H), 7.31 (t, *J* = 7.4 Hz, 2H), 7.24 (t, *J* = 7.3 Hz, 1H), 7.09 (d, *J* = 6.9 Hz, 2H), 5.32–5.13 (m, 2H), 4.78 (d, *J* = 16.8 Hz, 1H), 4.12–3.94 (m, 2H), 2.39 (s, 3H), 1.10 (t, *J* = 7.1 Hz, 3H).^13^C NMR (101 MHz, DMSO-*d*_6_) δ 165.36, 152.65, 150.11, 150.07, 138.59, 138.30, 130.17 (2C), 128.73, (2C) 126.98, 126.10 (2C), 111.96 (2C), 102.61, 59.86, 51.43, 44.79, 16.10, 14.03. HRMS (ESl): caled for C_21_H_20_Br_2_N_2_O_4_[M+Na]^+^: 546.9662, found: 546.9611.

### 2.4. Biological Evaluation

#### 2.4.1. Cell and Viruses

PK-15 cell line was purchased from the American Type Culture Collection (ATCC, Manassas, VA, USA). The porcine kidney epithelial cell line PK-15 was maintained in our laboratory and routinely cultured and passaged in DMEM supplemented with 10% fetal bovine serum. The pseudorabies virus strain PRV-QXX was kindly provided by Prof. Yongtao Li (College of Veterinary Medicine, Henan Agricultural University). The recombinant pseudorabies virus expressing green fluorescent protein (PRV-GFP) was generously provided by Prof. Hanzhong Wang (Wuhan Institute of Virology, Chinese Academy of Sciences). The recombinant vesicular stomatitis virus expressing green fluorescent protein (VSV-GFP) was preserved in our laboratory.

#### 2.4.2. Cell Viability Assay

PK-15 cells were seeded in 96-well plates and cultured overnight. The medium was then replaced with fresh medium containing the indicated concentrations of the test compounds (0, 0.2, 0.6, 2, 6, 20, and 60 μg/mL), with an equivalent volume of DMSO used as the vehicle control. After 24 h of treatment, CCK-8 reagent (DingGuo, Beijing, China) was added to each well, followed by incubation at 37 °C for 2 h. Absorbance at 450 nm was measured using a VARIOSKAN FLASH microplate reader (Thermo Fisher Scientific, Waltham, MA, USA).

#### 2.4.3. Flow Cytometry Assay

PK-15 cells were seeded in 24-well plates and cultured overnight. The cells were pretreated with medium containing the test compounds at 0, 0.2, 0.6, 2, 6, and 20 μg/mL for 6 h. Subsequently, cells were infected with PRV-GFP or VSV-GFP at an MOI of 0.01 for 1 h. After viral adsorption, the inoculum was removed and replaced with maintenance medium containing the corresponding concentrations of the compounds. Cells were further incubated for 24 h, harvested by trypsinization, and analyzed for GFP-positive cells by flow cytometry using a CytoFLEX instrument (BECKMAN COULTER, Miami, FL, USA). All data were analyzed using CytExpert 2.5.0.77 (Beckman Coulter, Miami, FL, USA). 

#### 2.4.4. RT-qPCR

PK-15 cells were seeded in 35 mm dishes and cultured overnight. The medium was replaced with medium containing 6 μg/mL of the compound for 6 h. The **4bf**-pretreated group was then infected with PRV-QXX (MOI = 0.01), and the **4ce**-pretreated group with VSV-GFP (MOI = 0.01). Cells were collected at 0, 2, 4, 6, 12, and 24 h post-infection. Total RNA was extracted using TRIzol Reagent (TaKaRa, Kyoto, Japan) and reverse-transcribed into cDNA with a PrimeScript RT Reagent Kit (TaKaRa, Kyoto, Japan) according to the manufacturer’s instructions. qRT-PCR was conducted in triplicate using SYBR Premix Ex Taq (TaKaRa, Kyoto, Japan). Relative gene expression was normalized to β-actin and calculated using the 2^−ΔΔCt^ method. Primer sequences are listed in Table 2.

#### 2.4.5. Western Blotting

Cells were washed twice with cold PBS on ice and lysed in RIPA buffer containing protease and phosphatase inhibitors.

(HY-K0010, HY-K0022; MedChemExpress, Monmouth Junction, NJ, USA). Lysates were centrifuged, and protein concentrations were determined using a BCA kit (BCA01, DingGuo, Beijing, China). Proteins were mixed with loading buffer, denatured at 99 °C, and separated by SDS-PAGE. After transfer to PVDF membranes (ISEQ00010, Millipore) and blocking with 5% nonfat milk (A600669, Sangon, Shanghai, China) for 1 h, membranes were incubated with primary antibodies (anti-PRV gB, anti-GFP, anti-β-actin) overnight at 4 °C, followed by HRP-conjugated secondary antibody for 1 h at room temperature, with washes after each step. Signals were visualized using Luminata Crescendo Western HRP Substrate (WBLUR0500, Millipore, Billerica, MA, USA) on a GE AI600 imaging system. All reagents and antibodies were stored at −20 °C.

#### 2.4.6. TCID_50_ Assay

PK-15 cells were seeded in 96-well plates and cultured overnight. The virus was tenfold serially diluted from 10^−1^ to 10^−12^, with eight replicates per dilution. After 1 h adsorption at 37 °C with 5% CO_2_, the medium was replaced with maintenance medium. Cytopathic effects (CPE) were observed and recorded for 5 consecutive days, and the TCID_50_ was calculated using the Reed–Muench method.

#### 2.4.7. Mouse Assay

All animal experimental protocols were approved by the Animal Care and Use Committee of Henan Agricultural University (HNND2025010811). Female 6-week-old Kunming mice were obtained from the Center of Experimental Animal of Zhengzhou University (Zhengzhou, China) and maintained under SPF conditions in accordance with the Guide for the Care and Use of Laboratory Animals and Henan Agricultural University ethical regulations. Mice were euthanized under isoflurane anesthesia followed by cervical dislocation.

For toxicity assessment, mice (*n* = 3 per group) received intraperitoneal injections of DMSO or compounds at 0, 5, 10, 15, 20, or 25 mg/kg (100 μL per dose) on days 1, 3, 5, 7, 9, and 11. Body weight and clinical signs were monitored daily. On day 12, mice were euthanized, and heart, liver, spleen, lung, kidney, and brain were collected and fixed in 4% PFA.

For antiviral evaluation, fifteen 6–8-week-old female SPF C57BL/6 mice were randomly assigned to Mock, PRV, or PRV + **4bf** groups (*n* = 5 each). Mock mice received DMSO intraperitoneally on days −1 and 0. PRV mice were intranasally challenged with PRV-QXX (3 × 10^3^ TCID_50_/mouse) on day 0. PRV + **4cf** mice received 20 mg/kg compound on day −1 and again with virus on day 0. Clinical signs were monitored daily, and lungs were collected in 4% PFA on day 7 post-infection.

#### 2.4.8. Tissue Processing and H&E Staining

Mouse tissues were fixed in 4% paraformaldehyde and trimmed to approximately the size of a soybean, followed by overnight washing. Fixed tissues were dehydrated through a graded ethanol series (70–100%), cleared with xylene, and embedded in paraffin. Sections (4 μm thick) were deparaffinized, rehydrated through a graded ethanol series, stained with hematoxylin and eosin (HE), cleared again with xylene, and mounted for histological observation under a light microscope.

#### 2.4.9. Statistical Analysis

All experiments were independently repeated at least three times. Statistical analyses were performed using GraphPad Prism 9.0. Comparisons between groups were conducted using unpaired *t*-tests. Differences were considered not significant (ns) or significant at * *p* < 0.05, ** *p* < 0.01, and *** *p* < 0.001. Graphs were prepared using GraphPad Prism 9, Microsoft Excel, and Adobe Illustrator.

## 3. Results

### 3.1. Chemistry

**Figure 2:**
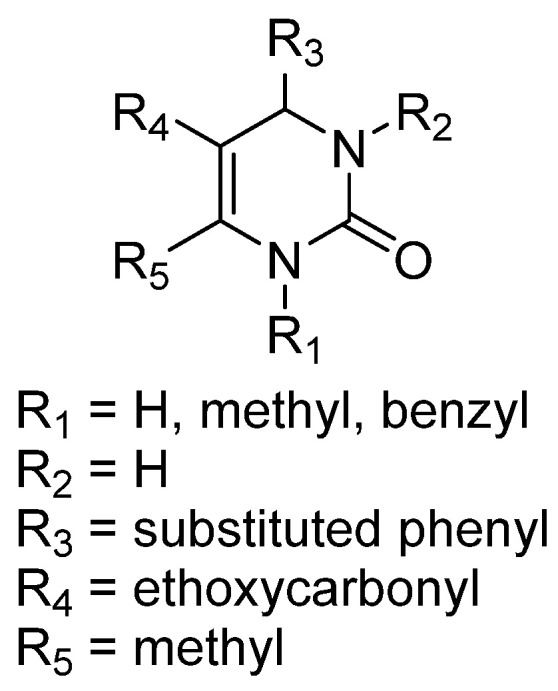
Design strategy of the target compounds.

The design strategy of the target compounds is illustrated in Figure 2. Based on the 3,4-dihydropyrimidin-2(1*H*)-one core, structural modifications were introduced at three positions: R_1_, R_3_, and R_4_. R_1_ was varied by reacting different urea derivatives to obtain H, methyl, or benzyl substituents; R_3_ was modified using substituted benzaldehydes bearing electron-withdrawing or electron-donating groups (e.g., fluorine, chlorine, bromine, methyl, hydroxyl, or methoxy) to expand the aromatic moiety; R_4_ was introduced via reaction with β-keto esters of varying chain lengths. Considering that the alkoxy chain length has limited impact on biological activity, ethyl acetoacetate was employed as the common reaction substrate in all experiments.

The synthetic route and structures of synthesized compounds are presented in Table 1. Three urea derivatives (compound **1**) were condensed with substituted benzaldehydes (compound **2**) to afford imine intermediates, which subsequently underwent nucleophilic addition with ethyl acetoacetate (compound **3**) in its enol form. By varying the starting materials, a series of 3,4-dihydropyrimidin-2(1*H*)-one derivatives bearing diverse substituents at R_1_, R_3_, and R_4_ was successfully obtained, providing a platform for further biological evaluation.

### 3.2. Biological Activity

#### 3.2.1. Cell Viability

The cytotoxicity of the target compounds toward PK-15 cells was evaluated using the CCK-8 assay. PK-15 cells were seeded into 96-well plates and cultured overnight, followed by treatment with various concentrations of the compounds (0, 0.2, 0.6, 2, 6, 20, and 60 μg/mL) for 24 h, with DMSO serving as the vehicle control. After incubation with CCK-8 reagent for 2 h, absorbance was measured, and the half-maximal cytotoxic concentration (CC_50_) values were calculated.

The 3,4-dihydropyrimidin-2(1*H*)-one derivatives exhibited low cytotoxicity within the tested concentration range (0–60 μg/mL). Among them, compounds **4bd**, **4bf**, and **4ab** displayed relatively lower cytotoxicity, with CC_50_ values of 280, 270.02, and 224.89 μg/mL, respectively, indicating favorable safety profiles in PK-15 cells (Table 3).

#### 3.2.2. Antiviral Activity

The antiviral activities of the 3,4-dihydropyrimidin-2(1*H*)-one derivatives against PRV and VSV were determined by flow cytometry. PK-15 cells were pretreated with different concentrations of the compounds and then infected with PRV-GFP or VSV-GFP (MOI = 0.01) for 1 h. After adsorption, the medium was replaced with maintenance medium containing the corresponding compounds, and cells were incubated for an additional 24 h. Viral replication was quantified by flow cytometry, and IC_50_ values were calculated.

Within the concentration range of 0–20 μg/mL, several compounds showed inhibitory effects on viral replication. For PRV, the IC_50_ values of compounds **4ci**, **4ad**, and **4ch** were 0.1749, 0.3351, and 0.5289 μg/mL, respectively. For VSV, the IC_50_ values of compounds **4al**, **4ci**, and **4cg** were 0.41, 0.5925, and 4.6737 μg/mL, respectively (Table 3).

#### 3.2.3. Selectivity Index

The selectivity index (SI = CC_50_/IC_50_) was calculated to further assess the antiviral activity of the 3,4-dihydropyrimidin-2(1*H*)-one derivatives by integrating cytotoxicity and inhibitory efficacy. The corresponding data are presented in Table 2.

In PRV-infected PK-15 cells, compounds **4aa**, **4ad**, and **4bf** exhibited SI values of 124.7, 133.4, and 243.0, respectively. In VSV-infected PK-15 cells, compounds **4al**, **4be**, and **4ce** showed SI values of 182.9, 181.1, and 196.4, respectively. Among these, **4bf** displayed the highest SI against PRV, whereas **4ce** showed the highest SI against VSV. Based on these results, compounds **4bf** and **4ce** were selected for further evaluation.

#### 3.2.4. In Vitro Antiviral Study of Compounds **4bf** and **4ce**

**Figure 3:**
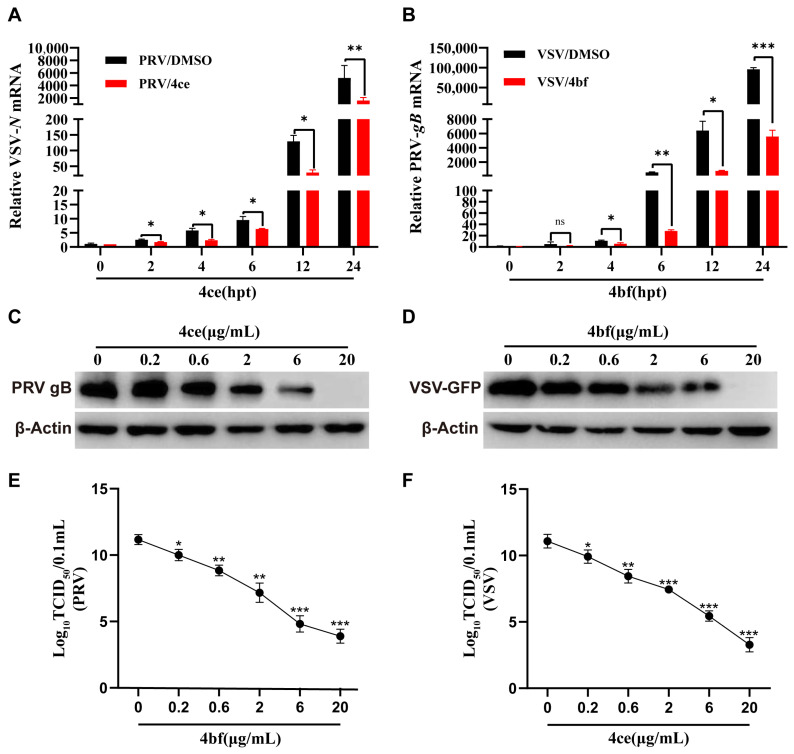
**qRT-PCR, TCID_50_ assay, and Western blot analysis were performed to evaluate the antiviral activity of the compounds.** (**A**,**B**) PK-15 cells were infected with PRV or VSV and treated with **4bf** or **4ce** (6 μg/mL), respectively. PRV-gB and VSV-N mRNA levels were measured by RT-qPCR. (**C**,**D**) Progeny virus titers were determined by the TCID_50_ assay. (**E**,**F**) PRV-gB and VSV-GFP protein levels were detected by Western blot, with β-actin as the loading control. * *p* < 0.05, ** *p* < 0.01, *** *p* < 0.001; ns, not significant.

To assess antiviral activity at the transcriptional level, the mRNA levels of PRV-gB and VSV-N were measured by RT-qPCR. PK-15 cells were pretreated with **4bf** or **4ce** (6 μg/mL) and then infected with PRV-QXX or VSV-GFP (MOI = 0.01), respectively. The expression levels of PRV-gB and VSV-N in the control group increased over time, whereas treatment with **4bf** markedly reduced PRV-gB mRNA levels and treatment with **4ce** significantly decreased VSV-N mRNA levels (Figure 3). The effect of the compounds on progeny virus production was further evaluated using the TCID_50_ assay. Increasing concentrations of **4bf** and **4ce** resulted in a clear reduction in PRV and VSV titers, respectively (Figure 3). Viral protein expression was further analyzed by Western blot. The levels of PRV gB and VSV GFP proteins decreased with increasing concentrations of **4bf** and **4ce** (Figure 3). These results indicate that **4bf** inhibits PRV replication, whereas **4ce** suppresses VSV replication in vitro.

#### 3.2.5. In Vivo Safety Evaluation and Antiviral Activity of Compound **4bf**

**Figure 4:**
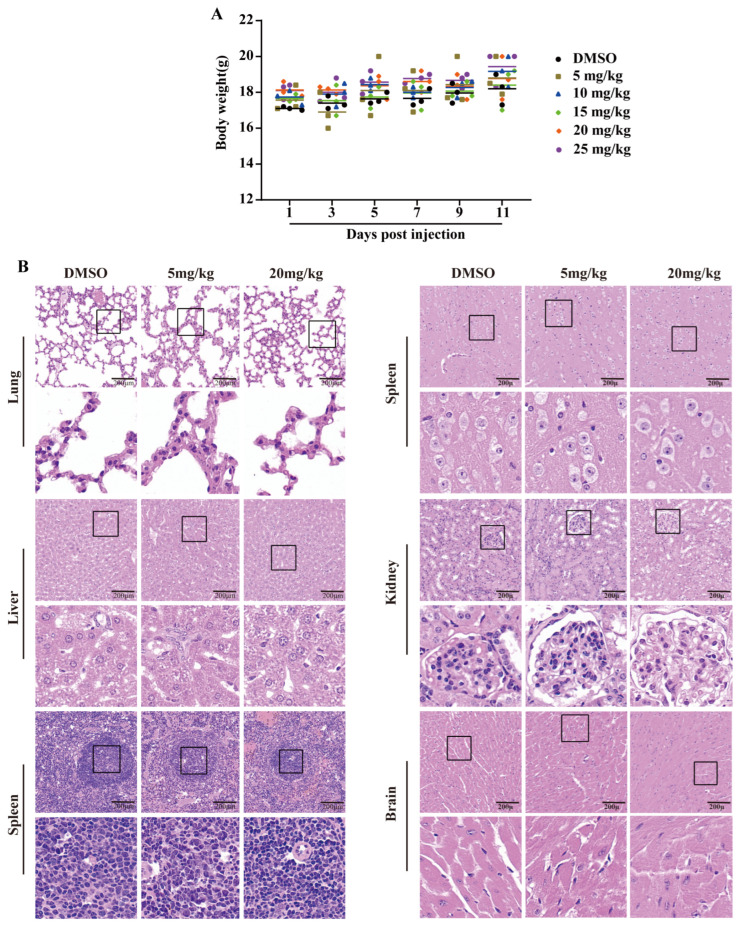
**In vivo safety evaluation of **4bf** in C57BL/6 mice.** (**A**) C57BL/6 mice (*n* = 3 per group) were intraperitoneally administered **4bf** at 0, 5, 10, 15, 20, or 25 mg/kg on days 1, 3, 5, 7, 9, and 11. Body weight was monitored throughout the experiment. No significant differences in body weight gain were observed among groups within the 0–25 mg/kg range. (**B**) On day 12, major organs (heart, liver, spleen, lung, kidney, and brain) were collected for H&E staining. No obvious histopathological alterations were observed in the 5 or 20 mg/kg groups compared with the DMSO control, indicating no apparent organ toxicity within the tested dose range.

To assess the in vivo safety of **4bf**, mice received intraperitoneal injections of **4bf** at 0, 5, 10, 15, 20, or 25 mg/kg on days 1, 3, 5, 7, 9, and 11, and body weight was monitored (Figure 4). Body weight gradually increased over time in all groups, with no significant differences compared with the control group. Histological analysis of major organs was conducted using H&E staining. Mice were sacrificed on day 12, and heart, liver, spleen, lung, kidney, and brain tissues were collected (Figure 4). No obvious histopathological abnormalities or tissue damage were observed at doses of 5 mg/kg and 20 mg/kg.

**Figure 5:**
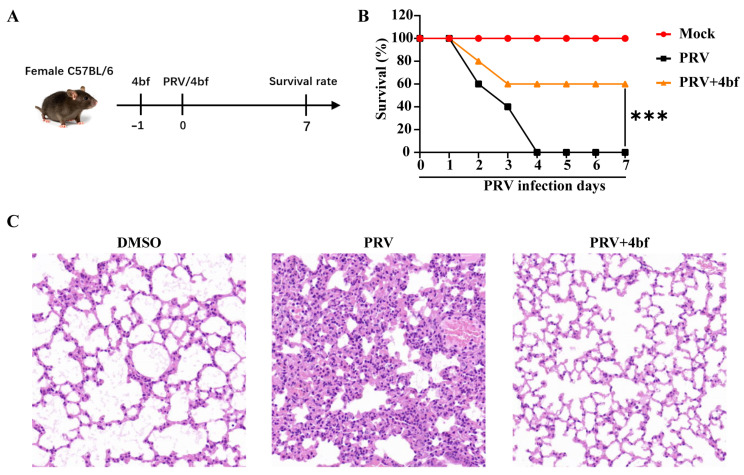
**Protective effect of 4bf against PRV infection in mice**. (**A**) Mice were intraperitoneally administered **4bf** (20 mg/kg) and then infected with PRV-QXX (3 × 10^3^ TCID_50_ per mouse). Survival was monitored throughout the experiment. (**B**) The PRV + **4bf** group showed a survival rate of 60%, whereas all mice in the PRV group died. (**C**) At day 7 post-infection, lung tissues were collected for H&E staining. Compared with the DMSO group, mice treated with **4bf** showed reduced inflammatory cell infiltration and milder lung tissue damage. *** *p* < 0.001.

The antiviral efficacy of **4bf** against PRV infection was then evaluated. Mice received 20 mg/kg **4bf** intraperitoneally, followed by intranasal infection with PRV-QXX (5 × 10^3^ TCID_50_ per mouse). Survival was monitored continuously (Figure 5). All the mice in the PRV group succumbed, whereas 60% of the mice in the PRV + **4bf** group survived. Lung tissues were collected on day 7 post-infection for H&E staining (Figure 5). Compared with the DMSO group, mice treated with **4bf** showed markedly reduced inflammatory cell infiltration and tissue damage.

## 4. Discussion

DHPM derivatives have been widely studied because of their diverse biological activities and modifiable scaffold. In this study, we synthesized a series of DHPM derivatives and evaluated their antiviral activity against PRV and VSV. Several compounds showed inhibitory effects on viral infection, among which **4bf**, **4ce**, and **4be** displayed relatively high selectivity indices. These results indicate that modification of the DHPM scaffold can improve antiviral activity.

The preliminary structure–activity relationship showed that antiviral activity was affected by both the aromatic substituents and the N-substitution pattern of the DHPM scaffold. Methoxy-containing compounds showed relatively good activity, especially against VSV. For example, **4ae**, **4be**, and **4ce** had high SI values against VSV, while **4bf** showed the strongest activity against PRV. This suggests that methoxy substitution may be beneficial for antiviral activity. The N-substitution pattern also influenced antiviral potency and selectivity. Compound **4aa** showed strong anti-PRV activity, whereas its N-methyl analogue **4ba** showed markedly reduced activity, indicating that N-methylation is not always favorable. However, in some substituted aromatic systems, N-methyl or N-benzyl substitution improved antiviral activity, as observed for **4bf** and **4ce**. Halogenated compounds also showed different activity profiles against PRV and VSV. For example, the brominated compound **4al** showed strong activity against VSV but weak activity against PRV, while some chlorinated compounds, such as **4ci** and **4bi**, showed improved activity against PRV or VSV. By contrast, compounds with multiple hydroxyl groups did not consistently show enhanced activity. These results indicate that methoxy substitution, halogen substitution, and suitable N-substitution are important factors affecting the antiviral activity of DHPM derivatives.

Further antiviral assays showed that the active compounds inhibited viral replication, reduced viral titers, and decreased viral protein expression and gene transcription. These results indicate that their antiviral effects are related to the inhibition of viral replication. Among these compounds, **4bf** was further evaluated in vivo. Compound **4bf** reduced PRV-induced pulmonary inflammation and tissue damage and improved the survival of infected mice, without obvious effects on normal growth. These results support the antiviral activity of **4bf** both in vitro and in vivo.

This study also has some limitations. First, although the antiviral activity of these DHPM derivatives was demonstrated, their exact antiviral targets and molecular mechanisms were not investigated. Further studies are needed to determine whether these compounds directly act on viral components or regulate host antiviral responses. Second, this study mainly focused on the preliminary evaluation of this class of compounds, and known antiviral drugs were not included as reference controls. In future studies, appropriate reference antiviral agents will be included for direct comparison to further evaluate the antiviral potential of DHPM derivatives.

## 5. Conclusions

In conclusion, a series of 3,4-dihydropyrimidin-2(1H)-one derivatives were synthesized through a one-pot Biginelli reaction and characterized by ^1^H NMR, ^13^C NMR, and high-resolution mass spectrometry. Antiviral evaluation showed that several compounds inhibited PRV and VSV infection, among which **4bf**, **4ce**, and **4be** showed the most promising activity. Further experiments showed that these compounds inhibited viral replication, reduced viral titers, and decreased viral protein and gene expression. In vivo, compound **4bf** alleviated PRV-induced lung injury and improved the survival of infected mice. These results indicate that DHPM derivatives, especially compound **4bf**, may serve as promising scaffolds for the development of antiviral agents.

## Figures and Tables

**Table 2:** Primers used for gene cloning and RT-qPCR analysis.

Name	Sequence (5′-3′)
Q-Sus-β-actin-F	CTGAACCCCAAAGCCAACCGT
Q-Sus-β-actin-R	TTCTCCTTGATGTCCCGCACG
Q-Sus-PRV-gB-F	GGCATCGCCAACTTCTTCC
Q-Sus-PRV-gB-R	CCTCGTCCACGTCGTCCTC
Q-Sus-VSV-N-F	TGATAGTACCGGAGGATTGACGAC
Q-Sus-VSV-N-R	CCTTGCAGTGACATGACTGCTCTT

**Table 3:** CC_50_, IC_50_ and SI of compounds.

Compounds	CC_50_ (μg/mL)	PRV		VSV	
		IC_50_ (μg/mL)	SI	IC_50_ (μg/mL)	SI
**4aa**	67.37	0.54	124.73	16.09	4.18
**4ab**	224.8	15.97	14.08	5.12	43.88
**4ac**	75.35	1.94	38.80	10.49	7.18
**4ad**	44.73	0.33	133.48	139.2	0.32
**4ae**	182.8	10.43	17.53	1.49	122.2
**4af**	67.35	52.05	1.29	8.34	8.07
**4ag**	165.8	18.89	8.77	25.28	6.55
**4ah**	85.13	22.08	3.85	64.38	1.32
**4ai**	86.85	9.27	9.36	33.46	2.59
**4aj**	90	12.86	6.99	40.98	2.19
**4ak**	177.6	17.22	10.31	10.26	17.31
**4al**	75	11.17	6.71	0.41	182.9
**4am**	91.78	8.68	10.56	4.92	18.64
**4ba**	65.50	43.16	1.51	4.57	14.31
**4bb**	150.7	99.81	1.51	6.96	21.64
**4bc**	175.7	6.61	26.57	17.52	10.02
**4bd**	280.0	43.84	6.38	>100	0.09
**4be**	190.9	1.53	124.2	1.05	181.1
**4bf**	270.0	1.11	243.0	4.14	65.08
**4bg**	81.13	42.71	1.89	594	0.13
**4bh**	167.9	>100	1.59	>100	0.59
**4bi**	94.19	15.24	6.18	1.07	87.70
**4bj**	89.96	8.15	11.03	48.25	1.86
**4bk**	68.36	13.69	4.99	>100	0.66
**4bl**	135.4	6.50	20.82	9.60	14.09
**4ca**	68.26	2.90	23.51	6.78	10.06
**4cb**	65.63	12.39	5.29	1.56	42.01
**4cc**	31.10	0.81	38.22	4.62	6.72
**4cd**	9.36	0.91	10.23	11.38	0.82
**4ce**	147.1	94.50	1.55	0.74	196.4
**4cf**	40.26	0.86	46.46	0.69	58.07
**4cg**	74.69	24.81	3.01	0.67	110.8
**4ch**	42.23	0.52	79.84	402	0.10
**4ci**	18.96	0.17	108.4	0.59	32
**4cj**	188.2	39.14	4.80	3.68	51.05
**4cm**	125.2	2.56	48.75	1.54	80.95
**4aa**	67.37	0.54	124.73	16.09	4.18
**4ab**	224.8	15.97	14.08	5.12	43.88
**4ac**	75.35	1.94	38.80	10.49	7.18
**4ad**	44.73	0.33	133.48	>100	0.32
**4ae**	182.8	10.43	17.53	1.49	122.2
**4af**	67.35	52.05	1.29	8.34	8.07

## Data Availability

The original contributions presented in this study are included in the article. Further inquiries can be directed to the corresponding authors.
